# Profile of Heavy Metals and Antioxidant Defense in the Muscle Tissues of Pigeons (*Columba livia* f. *urbana*) from Anthropogenically Transformed Areas in the Pomeranian Region (Northern Poland)

**DOI:** 10.1007/s00244-021-00825-3

**Published:** 2021-03-15

**Authors:** Natalia Kurhaluk, Halyna Tkachenko, Tomasz Hetmański, Agnieszka Włodarkiewicz, Vladimir Tomin

**Affiliations:** 1 Department of Biology, Institute of Biology and Earth Sciences, Pomeranian University in Słupsk, Arciszewski Str. 22b, 76-200 Słupsk, Poland; 2 Department of Earth Sciences, Institute of Biology and Earth Sciences, Pomeranian University in Słupsk, Słupsk, Poland; 3 Department of Physics, Institute of Science and Technology, Pomeranian University in Słupsk, Słupsk, Poland

## Abstract

Pigeons can be successfully used as bioindicators of a contaminated environment. We studied the relationship between the functioning of the pro/antioxidant balance in muscle tissues (skeletal muscle and cardiac tissues) of pigeons (*Columba livia* f. *urbana*) living in areas with different levels of pollution (Pomeranian Voivodeship, Northern Poland). The current study demonstrated the impact of the environment with preferential high Pb contamination in soil and feathers of pigeons on the formation of adaptive redox mechanisms in muscle tissues. An increase in the intensity of lipid peroxidation (estimated by the TBARS level) accompanied by enhancement of the oxidative modification of proteins (aldehydic and ketonic derivatives) and an important decrease in the activity of antioxidant enzymes (SOD, CAT, and GR) in pigeon muscle tissue was observed. These changes in enzyme activities were dependent on the type of muscle tissue (skeletal muscle and cardiac tissues). Our results confirm the concept of the recalculation of the De Ritis ratio (AsAT/AlAT) in both types of muscles indicating the tendency to cardio- and hepatocellular damage and toxicity caused by heavy metals from the polluted environment.

From year to year, cities become living environments for an increasing number of people. The increased attention to the study of urban problems and the accumulation of numerous data on the adaptation of birds to live in close contact with humans have contributed to the formation of a special research discipline that studies the avian fauna of urbanized landscapes (Cui et al. 2018). Among the synanthropic birds in Poland's cities and around the world, pigeons (*Columba livia*) are probably the most ecologically and toxicologically important species, because they reflect the whole complex of interactions with the natural environment and the human-created environments (Kurhalyuk et al. 2007). The pigeon was one of the first birds involved in human agriculture. It represents a group of synanthropic birds, i.e., species that have lost the ability to exist outside anthropogenic landscapes (Nam et al. 2003, 2006). This group includes species that have migrated beyond the natural range of humans. The adaptive capacity of these birds manifesting itself in nesting, feeding, ontogenetic development, and daily activity allows them to adapt to the habitat and nutrition patterns in the anthropogenic landscape and to settle widely (Schilderman et al. 1997; Liu et al. 2010). Due to the plasticity of the species, the range of the synanthropic pigeon continues to expand even today.

The feral pigeon (*Columba livia* f. *urbana*) occurs in larger cities on almost all continents of the world (Johnston and Janiga 1995). Its nesting colonies often are concentrated in the center of towns and cities (Sacchi et al. 2002), and feeding communities are observed even in places with heavy traffic. Recent studies conducted by Hetmański (2007, 2011) indicate that the pigeon is a sedentary species, as the birds do not migrate from the city to other localities and show great attachment not only to the location but also to one breeding colony. The dispersion of urban pigeons to other breeding colonies within one city occurs only in young birds, and this feature disappears completely in adult individuals (Johnston and Janiga 1995; Baldaccini et al. 2000). This feature makes pigeons very good indicators of the pollution of the urban environment, as birds accumulate various harmful substances from a specific area of the city in their bodies, most often in the vicinity of the breeding colony (Lodenius and Solonen 2013; Cui et al. 2013, 2016; Grúz et al. 2019). The strong philopatry towards their feeding and breeding sites is an advantage in monitoring studies, as it allows investigation of the difference in the level of pollution occurring in different districts of the city (Dauwe et al. 2003).

Feral pigeons have been used as biomonitor species of organic-induced environmental pollution. González-Gómez et al. (2020) have assessed levels of polychlorinated biphenyls, organochlorine pesticides, polybrominated diphenyl ethers, organophosphate pesticides, polycyclic aromatic hydrocarbons, and pyrethroids in body feather samples of feral pigeons observing the significant differences in age, location, and gender of birds. Different internal organs of birds can be used for research, and eggs, feathers, or blood can be tested as well (Hoff Brait and Antoniosi Filho 2011; Frantz et al. 2012; Pei et al. 2017). Investigation results have also shown that urban pigeons have several times higher levels of heavy metals accumulated in their tissues than domestic pigeons (Kouddane et al. 2016).

Szpęgawa is a village located in the Pomeranian Voivodeship by the Voivodeship road No. 224 in northern Poland. Near the village, there is the Stanisławie road junction of the A1 motorway. The A1 motorway in Poland is located within the international route E75 in the trans-European transport corridor, and it is the only Polish motorway with a southern route. The A1 motorway is characterized by heavy traffic. Currently, it connects the Tricity (Gdańsk, Gdynia, Sopot), which is as a large metropolitan center, with the Czech border (D1 motorway). The problem on such highways as the A1 is that they are accessed from narrow single-lane roads. As a result, there are huge numbers of slow-moving cars waiting in line to enter the highway. A busy road leading from the east of the country to the A1 motorway runs through Szpęgawa and Stanisławie. Nearby areas, such as Szpęgawa village in rural areas and the Stanisławie road junction, experience the huge negative impact associated with the emission of pollutants into the atmosphere. This is also related to the increased volume of freight traffic on international highways as well as the growing popularity of auto tourism. The level of ecological safety defines these conditions as dangerous technogenic systems, because the incomplete and uneven combustion of fuel is the main cause of air pollution. It is suggested that only 15% of the fuel is used to drive a car, while 85% is emitted into the atmosphere as an aerosol mixture of fuel and combustion products.

Pigeons are grain-eating birds with a relatively weak dental system, swallow soil stones (called gastroliths). Gastroliths play an important role in the digestive processes of animals (Johnston and Janiga 1995). Such stones are purposefully swallowed and contribute to fragmentation of dense and fibrous food in the stomach. Therefore, both intensive traffic and heavy metal pollution near the location of pigeons can impact their welfare and health condition (Hoff Brait and Antoniosi Filho 2011).

The metabolic changes and oxidative stress in organisms caused by heavy metals in a degraded environment (with varying degrees of anthropopressure) include mainly lipid peroxidation processes, oxidative damage to proteins and DNA, and reduction of the capability of the antioxidative barrier. These changes are only the peak of the iceberg, because products of oxidative stress damage all classes of molecular components of cells. Numerous studies performed on animals and humans show that reactive oxygen species (ROS) play an important role in pathological processes and disorders of different origins (Patrick 2003, 2006).

The range of environmental factors, resulting from human activity, contained in water, inhaled air, or food and capable of inducing pathological free radical reactions is constantly expanding and exerts an impact on animals living in intensively changed areas. Birds of towns and cities are exposed to such effects (Hoff Brait and Antoniosi Filho 2011). Gene mutations induced in this way promote carcinogenesis and are examples of disorders caused by physical factors involving oxygen radicals (Patrick 2003, 2006).

The current study was designed to assess the influence of varied environmental-induced stress on pigeons living in different contaminated areas (Szpęgawa and Słupsk, Pomeranian Voivodeship, Northern Poland) on the oxidative parameters in the muscle tissue (intensity of lipid peroxidation, oxidatively modified proteins, total antioxidant capacity, activities of antioxidant enzymes) and contents of heavy metals. The main goals of the study were: (1) to determine whether the metal contents differ between soil and feathers of pigeons living in areas with different levels of anthropopressure; (2) to evaluate the relationship between heavy metals and oxidative stress biomarkers in the muscle tissues (skeletal and cardiac) of pigeons; (3) to estimate the relationship between markers of hepato- and cardiotoxicity in muscle tissues; (4) to characterize the trend of main effects (i.e., the type of environment and type of tissue) in the formation of oxidative stress biomarkers and element contents in the muscle tissue and the interaction of the environment and tissue effects simultaneously; (5) to evaluate important determinants and correlative and regressive dependences of the antioxidant defenses in different muscle tissues of pigeons.

## Materials and Methods

### Ethical Approval

The experiments were conducted by the Guidelines of the European Union Council and the current laws in Poland. The study was conducted with the consent of the Bioethics Committee of Gdansk University (Poland, License Number 44/2012).

### Study Area

**Fig. 1:**
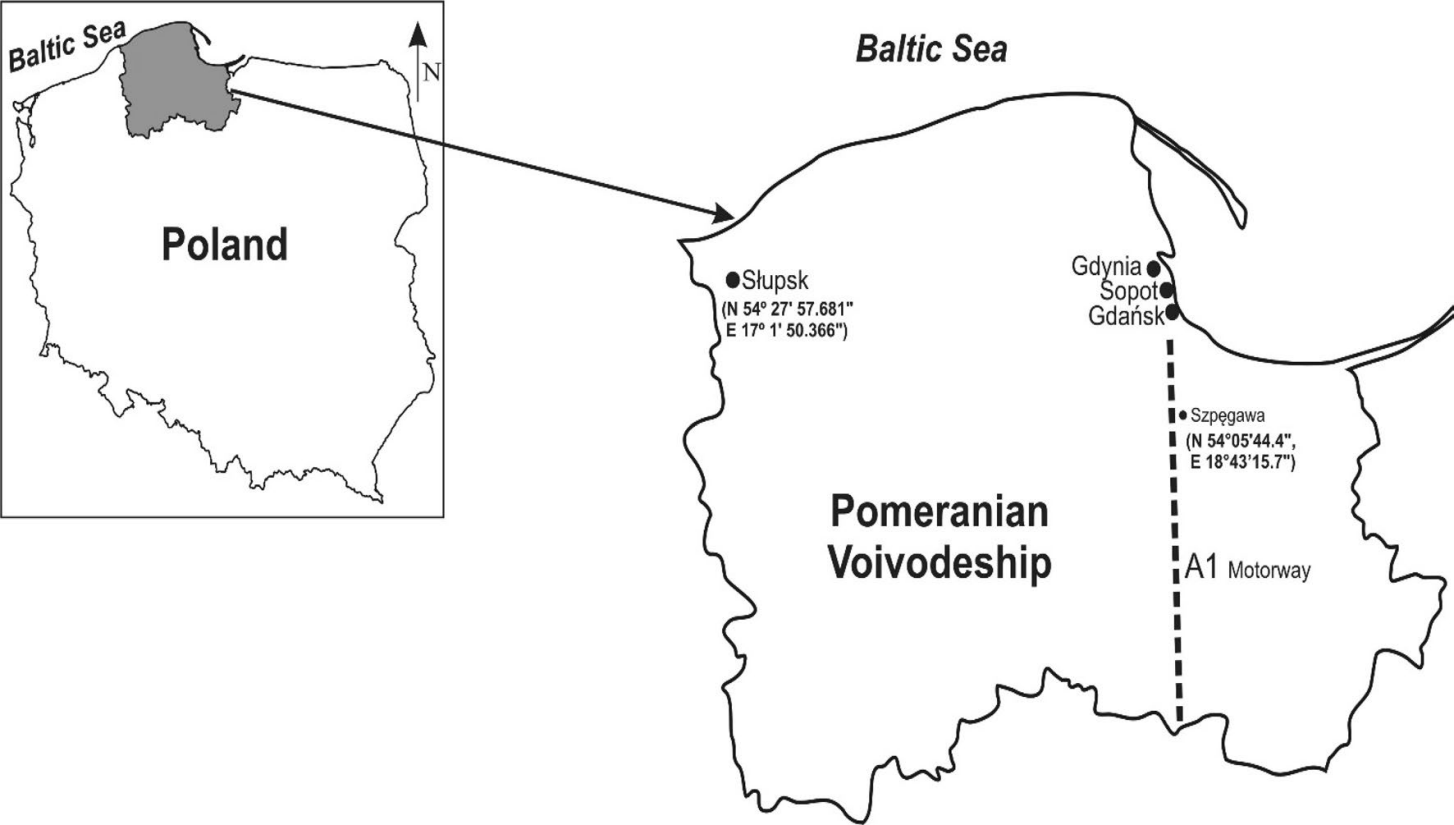
Map of the study area. Szpęgawa (N 54° 05′ 44.4", E 18° 43′ 15.7") located near the A1 motorway and Słupsk (N 54° 27′ 57.681" E 17° 1′ 50.366") in the Pomeranian Voivodeship are marked

The research was conducted in the Słupsk and Szpęgawa located in the Pomeranian Voivodeship, northern Poland (Fig. 1). Słupsk (N 54$^\circ$ 27′ 57.681" E 17$^\circ$ 1′ 50.366") is a city with 90,000 inhabitants located in the central part of Pomerania. In the central part of the city is the Old Market, a recreational and tourist center. At the same time, the Old Market has been a feeding ground for the largest flock of the urban pigeon (*Columba livia* f. *urbana*) with more than 300–400 individuals for many years. The first pigeons appeared in this town in the 1980s. The area where the urban pigeons feed is partly paved; the soil for the urban vegetation has been brought from the neighboring agricultural areas.

Szpęgawa is a village located approximately 120 km east of Słupsk. The village includes farms located on agricultural land. For the study of suburban pigeons, a farm located at the point with coordinates N 54° 05′44.4", E 18° 43′15.7" was selected. It is an old farm established after World War II. To harden the ground on the farm, loose slag (trail), i.e., a waste material from metallurgical production, was brought in the 80s of the past century. The farm also houses the breeding of urban pigeons originating from the pigeon population from Słupsk. The colony was established in 2007–2008 to conduct a series of experimental studies, the results of which have been published (Hetmański 2011).

### Characteristics of the Experimental Groups

Adult pigeons aged at least 1 year were the study material. These were not young birds, which can be easily distinguished from adults by the color of their plumage and the appearance of the nostril cere on the beak (Johnston and Janiga 1995). However, the exact age of the captured birds was unknown and cannot be determined by the external or internal features of the bird's body. Sexual maturation of pigeons begins already after they are 3–4 months old (Hetmański 2011). The sex of the pigeons captured for the study was determined according to the type of gonads (presence of testes or ovaries) only after decapitation. Among the birds captured in Słupsk, there were 7 females and 10 males, and in the sample from Szpęgawa, there were 7 females and 7 males. Identification of the sex of pigeons based on the type of gonads is the best method, as sexual dimorphism is poorly visible in this species. Identification of the sex of live pigeons requires long-term observation of bird behavior. The mean bodyweight of the pigeons was 398.70 ± 28.10 g in the Słupsk group (*n* = 17) and 409.80 ± 27.76 g (*n* = 14) in the Szpęgawa sample. The pigeons from Szpęgawa were slightly heavier than those caught in Słupsk, but the difference was not statistically significant (*p* = 0.281).

Muscle tissues (skeletal and cardiac) from 17 pigeons in Słupsk and 14 pigeons in Szpęgawa were used for the study. Hearts and skeletal muscles (*pectoralis* m.) were removed from the pigeons after decapitation. One pigeon was used for each preparation. Briefly, the hearts and skeletal muscles were excised, weighed, and washed in the ice-cold buffer. The minced tissue was rinsed to remove blood with cold isolation buffer and homogenized in a homogenizer H500 (POL-EKO, Poland) with a motor-driven pestle on ice. Homogenates were centrifuged at 3000 g for 15 min at 4 °C. The isolation buffer contained 180 mM KCl, 10 mM HEPES, 10 mM EGTA, and 0.5% bovine serum albumin; the pH value was adjusted to 7.3 with KOH. The suspension was then centrifuged for 5 min at 600 g at 0 °C.

Surface soil samples were collected from both sites to analyze the heavy metal content in the soil from which pigeons pick up gastroliths. In Słupsk, four soil samples (each sample was analyzed in 3 replicates) were taken from the Old Market square, where the largest flock of pigeons is located. In Szpęgawa, four soil samples (analysis of each sample was performed in 3 replications) were taken from an area between farm buildings where the birds receive food and water. We also sampled feathers from five adult birds (minimum 1 year old) in each place. Contour feathers were taken from the birds' backs.

### Reagents and Solutions

EDTA, HEPES, KCl, K_2_CO_3_, KH_2_PO_4_, EDTA, and 2-thiobarbituric acid were purchased from Sigma-Aldrich (Sigma-Aldrich Sp. z o.o, Poznan, Poland). The reagents were freshly prepared. All other reagents used were of analytical reagent grade.

### Biochemical Assays

Tissue homogenates were used for the determination of the levels of 2-Thiobarbituric acid reactive substances (TBARS), oxidative modification of proteins (OMP), antioxidant defense enzymes, and total antioxidant capacity (TAC). The Bradford method (1976) with bovine serum albumin as a standard was used for the quantification of proteins. Absorbance was recorded at 595 nm.

### 2-Thiobarbituric Acid Reactive Substances (TBARS) Assay

The level of lipid peroxidation was determined by quantifying the concentration of 2-thiobarbituric acid reacting substances (TBARS) with the Kamyshnikov (2004) method for determining the malonic dialdehyde (MDA) concentration. This method is based on the reaction of the degradation of the lipid peroxidation product, MDA, with 2-thiobarbituric acid (TBA) at high temperature and acidity to generate a colored adduct measured spectrophotometrically. The nmol of MDA per mg protein was calculated using 1.56·10^5^ mM^−1^ cm^−1^ as the extinction coefficient.

### Protein Carbonyl Derivative Assay

The assay of carbonyl derivative content of oxidative modification of proteins (OMP) was performed based on the spectrophotometric measurement of aldehydic (AD) and ketonic derivatives (KD) in the tissue samples. The rate of protein oxidative destruction was estimated from the reaction of the resultant carbonyl derivatives of amino acid reaction with 2,4-dinitrophenylhydrazine (DNFH) as described by Levine et al. (1990) and modified by Dubinina et al. (1995). DNFH was used for determining the carbonyl content in soluble and insoluble proteins. Carbonyl groups were determined spectrophotometrically from the difference in absorbance at 370 nm (aldehydic derivatives, OMP_370_) and 430 nm (ketonic derivatives, OMP_430_).

### Superoxide Dismutase Activity Assay

Superoxide dismutase (SOD, E.C. 1.15.1.1) activity in the supernatant was measured according to Kostiuk et al. (1990). SOD activity was assessed by its ability to dismutate superoxide produced during quercetin auto-oxidation in an alkaline medium (pH 10.0). The activity was expressed in units of SOD per mg of protein.

### Catalase Activity Assay

Catalase (CAT, E.C. 1.11.1.6) activity was determined by measuring the decrease in H_2_O_2_ in the reaction mixture with the method developed by Koroliuk et al. (1988). One unit of CAT activity was defined as the amount of the enzyme required for decomposition of 1 μmol of H_2_O_2_ per min per mg of protein.

### Glutathione Reductase Activity Assay

Glutathione reductase (GR, EC 1.6.4.2) activity in the tissue was measured according to the method described by Glatzle et al. (1974). The enzymatic activity was assayed spectrophotometrically by measuring NADPH(H^+^) consumption. In the presence of GSSG and NADPH(H^+^), GR reduces GSSG and oxidizes NADPH(H^+^), resulting in a decrease in the absorbance at 340 nm. The rate of NADPH oxidation was followed spectrophotometrically at 340 nm. Quantification was performed based on a molar extinction coefficient of 6.22 mM^−1^ cm^−1^ of NADPH. The GR activity was expressed as μmol NADPH(H^+^) per min per mg of protein.

### Glutathione Peroxidase Activity Assay

Glutathione peroxidase (GPx, EC 1.11.1.9) activity was determined by detecting the nonenzymatic utilization of GSH (the reacting substrate) at an absorbance of 412 nm after incubation with 5,5-dithiobis-2-nitrobenzoic acid (DTNB) according to the method proposed by Moin (1986). The rate of GSH reduction was followed spectrophotometrically at 412 nm. GPx activity was expressed as μmol GSH per min per mg of protein.

### Total Antioxidant Capacity Assay

The TAC level in the sample was estimated by measuring the TBARS level following Tween-80 oxidation. This level was determined spectrophotometrically at 532 nm by Galaktionova and co-workers (1998). The sample inhibited the Fe^2+^/ascorbate-induced oxidation of Tween-80, resulting in a decrease in the TBARS level. The level of TAC in the sample (%) was calculated for the absorbance of the blank.

### Alanine Aminotransferase (AlAT) and Aspartate Aminotransferase (AsAT) Activity Assay

AlAT and AsAT activity was analyzed spectrophotometrically with the standard enzymatic method described by Reitman and Frankel (1957). One unit of AsAT or AlAT was defined as the liberation of 1 μmol of pyruvate per hour at 37 °C incubation per 1 mg protein.

### Determination of Element Concentrations

The soil samples were collected at a depth of 1–3 cm. Then, the samples were aggregated and air-dried before storage and analysis. Each soil and feather sample was analyzed in three series. Between different readings, the soil sample was thoroughly mixed within the same bag. The results of three readings were averaged.

The concentrations of chemical elements were analyzed in the feather and soil samples with an x-ray fluorescence (XRF) analyzer at the Department of Physics, Pomeranian University in Słupsk (Poland). The XRF analyzer, model Sci Sps X-200 from Sci Sps, Inc., was used for determination of the concentrations of chemical elements in the samples. The analyzer is constructed to study elements in different samples, such as soil, alloys, precious metals, and some others.

The XRF (x-ray fluorescence) analyzer generates an x-ray beam that can be used for irradiating the sample. Interaction of the x-ray quanta with the analyzed sample causes characteristic x-ray emission from chemical elements in the sample. The analyses were conducted with an Rh target (50 kV, 600 μA) and polycapillary optics providing a spot size of 25 μm. The x-ray fluorescence signal was collected by two XFlash silicon drift detectors. They provide high spectral resolution of 135 eV measured on the full width at half maximum, FWHM, at 5.95 Mn K-alpha line. The detectors register the spectra of Roentgen fluorescence, or x-ray fluorescence, containing information about the presence of chemical elements and their concentrations. Commonly, the K and L series of x-ray fluorescence are used for identification of chemical elements, because they yield the best results. The detectors have an active area of 30 mm^2^ placed at 45° to the x-ray beam. The analyses were performed under vacuum (20 mbar), using a sampling step of 20 μm and 10 ms dwell time. The apparatus is factory calibrated with 37 standard elements, including all measurable pathfinders. The x-ray fluorescence hyperspectral data were processed using PyMca 5.1.3 (Solé et al. 2007) and Datamuncher (Alfeld and Janssens 2015) software. The device software uses either standard methods, such as basic parameters for the spectra of the given elements (we used this method in our measurements) or user-generated empirical calibration curves to relate the x-ray spectrum to the element concentrations.

The spectrometer can record sample information from an area of approximately 0.5 cm^2^, and the penetration range is from 5 μm to 5,000 μm, depending on the surface, density, and absorbency of the material. The x-ray tube and detector are located at the top of the front of the spectrometer. Additionally, there is a sensor underneath them to prevent accidental switching on of the measurement. There also is a camera in the measurement window that allows you to view the sample. The measuring area of the device is small.

An important factor limiting detection is moisture or water damage to the sample; therefore, the material was dried before analysis. When the sample is too far from the detector due to its nonuniformity or its density is too small, the measurement will not be started, because the device will inform about too few detector counts and will block the measurement. An ideal sample should be homogeneous, dry, and have a smooth surface. For our best results, the material was ground, and then samples were prepared.

The samples were made in special containers with a removable bottom made of polypropylene foil with a thickness of 6.0 μm. Of this type, the foil does not limit the energy emitted by the sample. (Soil: diameter 5 mm, height 10 mm, weight 800 mg; samples with feathers: diameter 5 mm, height 10 mm, weight 200 mg.) To obtain reliable results before each test, the analyser was factory calibrated by Compton normalization (EPA method 6200). The measuring time depends on the accuracy of interest. The entire period is divided into two phases (two time intervals). The first phase is responsible for the detection of heavy elements; the results of high accuracy are achieved in approximately 30 s. The voltage of the x-ray tube in this phase is 50 kV, additionally a filter that cuts off the influence of light elements is turned on. After the transition to the second phase, the voltage drops and the light element measurement begins. In our study, the measurement lasted 60 s for each sample.

It should be remembered that tests with the use of X-200 allow to determine the percentage share of a given metal in the total amount of metals in the tested sample with the accuracy ± 3%. The stability of the x-radiation intensity during the measurements was within the limits ± 2%. For a better understanding of the obtained data, the x-ray spectra of the samples also were displayed on the computer, which allowed for better verification of the data issued to the analyzers in an automatic regime.

### Statistical Analysis

The basic statistical analysis (significance of regression slopes, analysis of variance for significance between the localities and between tissues for metals and enzyme activity, distribution testing) was done using the STATISTICA 13.3 package (StatSoft, Krakow, Poland). The data were tested for homogeneity of variance using Levene’s test of equality of error variances. Normality was checked by the Kolmogorov–Smirnov test.

The results are expressed as mean ± standard deviation (SD). Significant differences among the means were measured using a multiple range test at min *P* < 0.05. Data not having a normal distribution were log-transformed. Student *t*-tests with 95% confidence intervals (α = 0.05) were applied to determine the significance of differences between element concentrations in the types of regions and the significance of differences in the element level in soil and feathers of birds and enzyme activity in tissues (skeletal muscles, heart) of birds from the different regions. The correlation of parametric values was based on Pearson’s regression analysis using the multiple regression module. The correlation and regression analysis comprised the correlation coefficient (r), regression equation, and significance of these dependencies (P). The arithmetic means of the concentrations of elements and enzyme activity in the muscle and heart tissues were estimated using two-way ANOVA. The use of multivariate significance tests of the main effects (type of the environment, type of tissue, and their combined effects) allowed determination of statistically significant relationships for all three values. In the model approach, to combine the impact of two factors (environment and tissues), we adopted a two-way classification model in the following form:

$${\text{X}}_{{{\text{ijk}}}} = \, \mu \, + \, \alpha_{{\text{i}}} + \, \beta_{{\text{j}}} + \left( {\alpha \beta } \right)_{{{\text{ij}}}} + \varepsilon_{{{\text{ijk}}}} ,$$

where X_ijk_ –value of the dependent variable, µ—mean, α_i_—main effect of the environment factor, β_j_—main effect of the tissue factor; (αβ)_ij_—effect of the interaction of the environment and tissue type factors; ε_ijk_—random experimental error.

We used the coefficients of multiple correlation analysis (R), the coefficient of determination (R^2^), and its corrected form reduced by random errors (R^2^ adjusted) in the data analysis for the description of the full model. We used the SS test to describe the share of all analyzed biomarkers of oxidative stress and biochemical parameters for assessment of the antioxidant barrier with the F test and its significance (Zar 1999).

## Results

### Metals

As shown in Table 1, the metal content in the soils in the areas studied differed significantly, i.e., a statistically significantly higher level of elements was observed in Szpęgawa compared with the results obtained from the Słupsk soil. This allowed us to classify this area as contaminated (Polluted area), because the level of metals except for Si, Ni, and Cu was statistically higher compared with the data from the Słupsk area. In the soil samples from Szpęgawa, the level of Al was higher by 121%, Ti by 23%, Mn by 242%, Fe by 15.5%, and Pb by 543.5% than in Słupsk (nonpolluted area). The lead level was fivefold higher in the soil from Szpęgawa. In the case of metals, such as Zn, Zr, and Si, significantly higher levels were observed in Słupsk than in Szpęgawa.

**Table 1:** Mean concentrations of metals (g/kg) ± standard deviation (SD) in soil collected from the different areas (polluted area—Szpęgawa; nonpolluted area—Słupsk, Pomeranian region, Northern Poland)

Elements	Polluted area	Nonpolluted area	*p*	F
Al	76.55 ± 31.53	36.68 ± 14.03	0.001	5.049
Si	78.87 ± 40.27	218.74 ± 56.60	0.000	1.976
Ti	36.23 ± 5.87	28.18 ± 5.79	0.003	1.026
Mn	11.65 ± 2.22	3.31 ± 2.42	0.000	1.183
Fe	720.78 ± 18.12	627.88 ± 76.95	0.001	18.02
Ni	5.41 ± 1.52	4.39 ± 1.11	0.075	1.866
Cu	4.18 ± 0.72	3.93 ± 1.29	0.580	3.182
Zn	10.29 ± 1.24	24.25 ± 9.02	0.000	52.94
Zr	26.04 ± 9.57	48.33 ± 20.98	0.003	4.812
Pb	28.74 ± 9.21	4.46 ± 3.17	0.000	8.434

*p* significant site-dependent differences in metal levels; *F* variance level

Data were collected and analyzed from five independent samples. Student *t*-tests with 95% confidence intervals (*p* = 0.05) were applied to determine the significance of differences between element concentrations in the different areas and the significance of differences in element levels in the soil

The next stage of our research was to determine metal levels in the feathers of birds living in different areas. The content of elements in pigeon feathers was ambiguous. Pigeons from the polluted area had statistically significant higher levels of Si and Pb in their feathers as well as low levels of Fe, Cu, and Zn compared with those from the Słupsk area. The analysis of metal content in the soil and feathers of birds showed different results, but because the lead content in both soil and feathers was significantly higher, we suggested that there were alterations in the metabolism of birds caused by the pronounced lead accumulation.

### Pro/antioxidant Balance

**Fig. 2:**
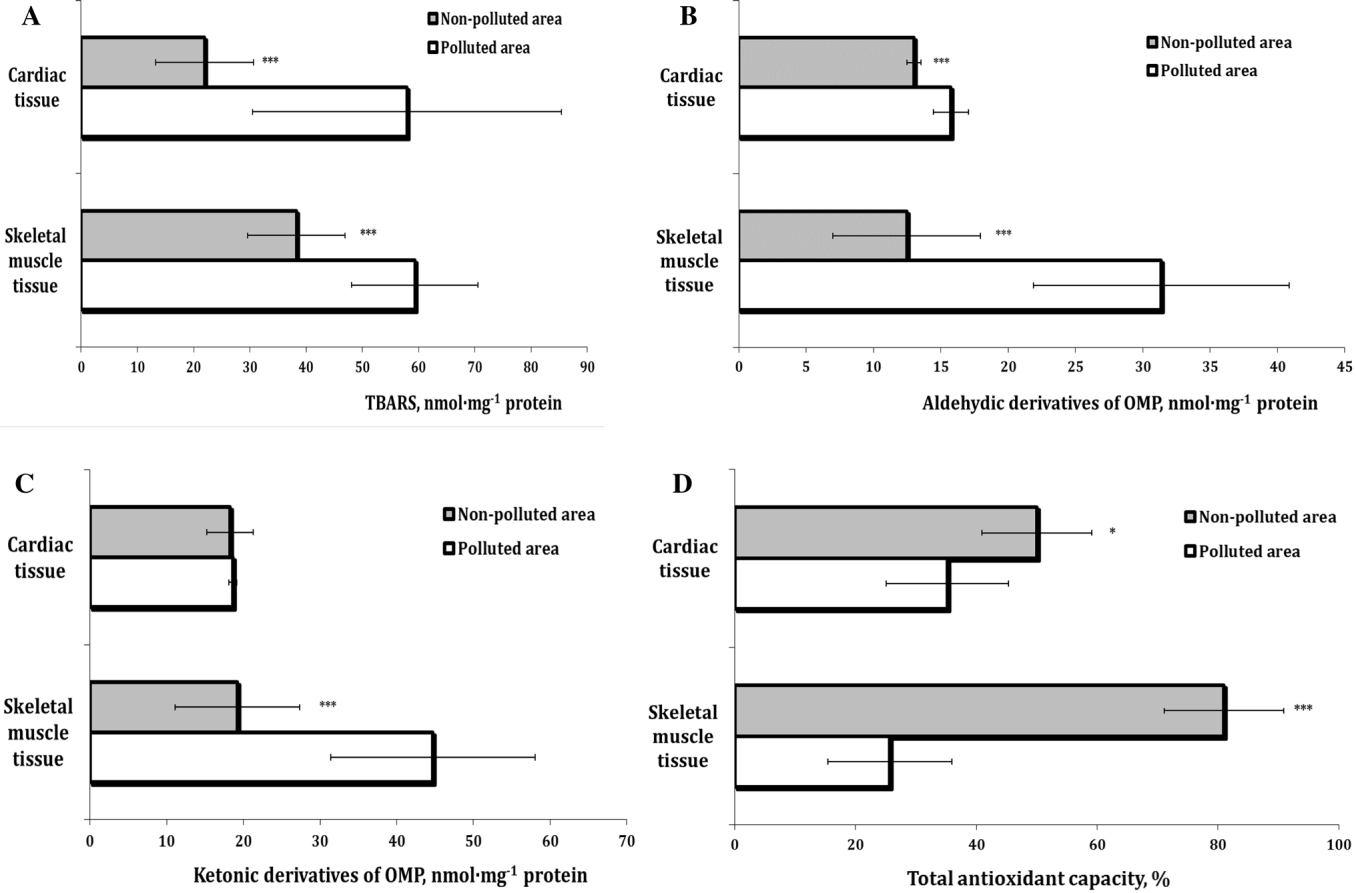
Content of TBARS (nmol MDA·mg^−1^ protein, **a**), aldehydic derivatives (AD, nmol·mg^−1^ protein, **b**), and ketonic derivatives (KD) of oxidatively modified proteins (OMP) (nmol·mg^−1^ protein, **c**), and total antioxidant capacity (TAC, %, **d**) in pigeon muscle tissues (skeletal muscle and cardiac tissues) sampled from the different areas (polluted area—Szpęgawa; nonpolluted area—Słupsk, Pomeranian region, Northern Poland). *p < 0.05; **p < 0.01; ***p < 0.001

Lipid peroxidation reactions caused by reactive oxygen species (ROS) result in the formation of many degradation products. One of the final products of these processes, i.e., malonic dialdehyde, reacts with 2-thiobarbituric acid to form 2-thiobarbituric acid reactive substances (TBARS). MDA is currently the most commonly used lipid peroxidation marker; its level indicates pathological changes in the organism. Therefore, we decided to analyze its level in our study first (Fig. 2A). The highest level of TBARS products in the skeletal and cardiac muscles in pigeons from the polluted (P) area compared with that from the nonpolluted (NP) region was observed. Noteworthy, higher intensity of lipid peroxidation in the cardiac tissue than in the muscle tissue was detected.

Oxidative stress effects at Pb intoxication have harmful consequences on skeletal and cardiac muscle tissues, i.e., oxidation of highly reactive free radicals and potential protein damage. Therefore, we estimated the levels of protein carbonyl derivatives (aldehydic and ketonic derivatives, respectively) and total antioxidant capacity (TAC) in the pigeon muscle tissues. Our results show a significant effect of Pb exposure in the two analyzed types of tissues. These changes were accompanied by an increase in the levels of oxidatively modified proteins (Figs. 2b, c). The current study revealed a tendency to decrease the TAC level in muscle tissues in both types. It is known that the ability to scavenge ROS in an organism can be assessed by TAC determination. The TAC indicator is frequently used to assess the antioxidant status of biological samples and can evaluate the antioxidant response against free radicals produced in a given disease (Rubio et al. 2016). In our study, the TAC level was decreased statistically in the birds from the polluted area (Szpęgawa village).

It should be noted that the negative pollution-induced changes in the level of oxidatively modified proteins (151% for aldehydic derivatives and 132% for ketonic derivatives) and total antioxidant capacity (43% and 211% in the cardiac and muscle tissues, respectively) were more pronounced in the percentage ratio in the skeletal muscle tissue than in the cardiac tissue.

**Fig. 3:**
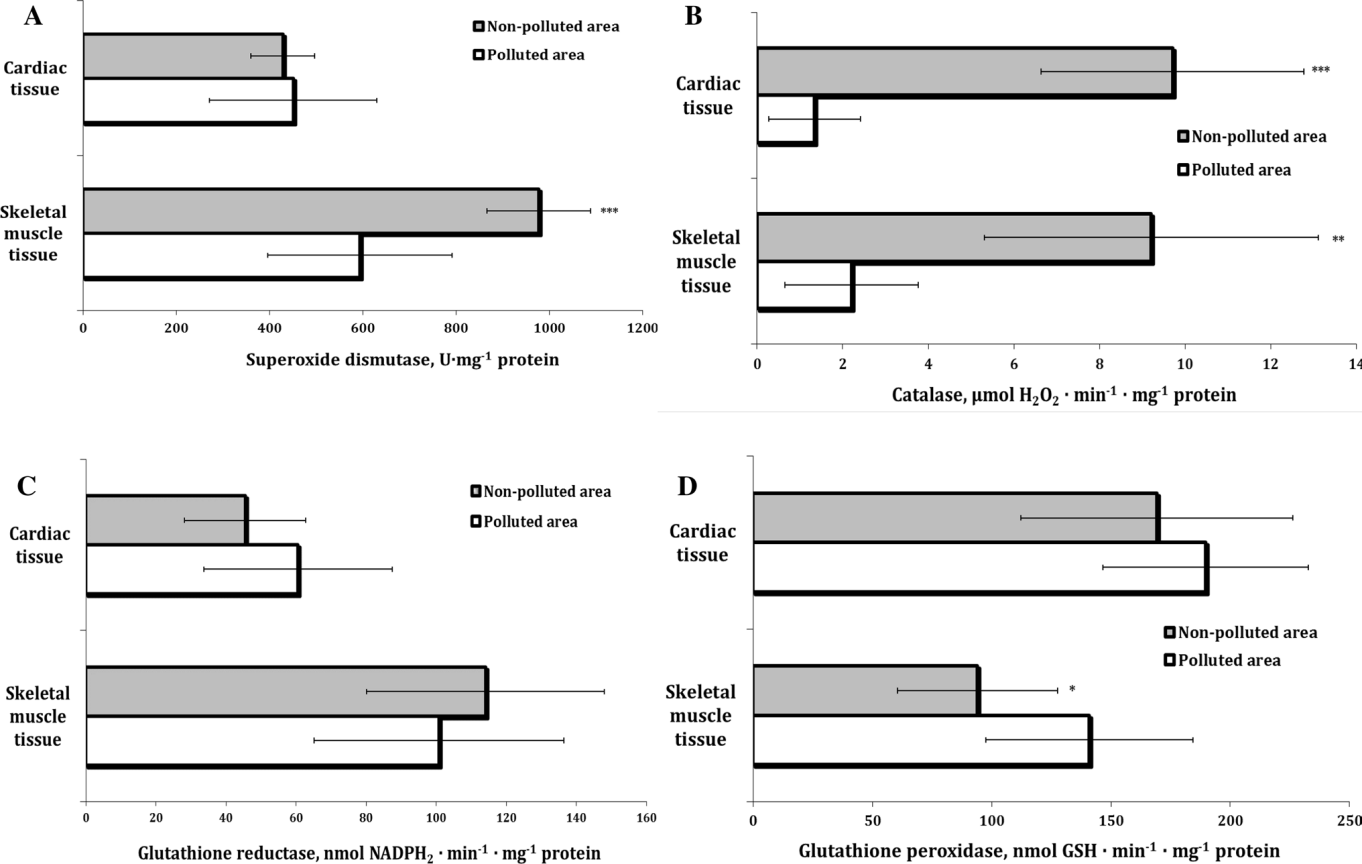
Activities of superoxide dismutase (SOD, U·mg^−1^ protein, **a**), catalase (CAT, μmol·min^−1^· mg protein^−1^, **b**), glutathione reductase (GR, μmol min^−1^ mg protein^−1^, **c**), and glutathione peroxidase (GPx, μmol min^−1^ mg protein^−1^, **d**) in pigeon muscle tissues (skeletal muscle and cardiac tissues) sampled from the different areas (polluted area—Szpęgawa, nonpolluted area—Słupsk, Pomeranian region, Northern Poland). *p < 0.05; **p < 0.01; ***p < 0.001

In our study, the activities of enzymes associated with the first and second lines of antioxidant defenses, i.e., SOD and CAT, exhibited changes presented in Figs. 3a, b. The lowest SOD activity was observed in the skeletal muscles of pigeons from the polluted area. The CAT activity exhibited a similar tendency to that found for SOD, i.e., a tendency to decrease in the pigeons from the polluted environment. It should be noted that the catalase activity in the cardiac and muscle tissue of pigeons from the polluted areas exhibited a statistically significant decrease. In the percentage ratio, there was a threefold and sixfold decrease in the skeletal muscle tissue and cardiac tissue, respectively, compared with the birds from the nonpolluted areas.

The activities of enzymes associated with glutathione metabolism, i.e., GR and GPx, in the different muscle tissues are presented in Figs. 3c, d. The highest activity of GPx was observed in the skeletal tissue of pigeons from the polluted area (Table 2).

**Table 2:** Mean concentrations of metals (g/kg) ± standard deviation (SD) in feathers of pigeons sampled from the different areas (Polluted area—Szpęgawa, Non-polluted area—Słupsk, Pomeranian region, Northern Poland)

Elements	Polluted area	nonpolluted area	*p*	F
Al	52.75 ± 9.99	48.52 ± 13.31	0.333	1.772
Si	894.59 ± 16.08	861.52 ± 32.93	0.002	4.195
Fe	11.88 ± 6.99	20.18 ± 9.86	0.013	1.987
Ni	2.11 ± 0.58	2.54 ± 0.69	0.078	1.440
Cu	4.55 ± 1.33	5.57 ± 1.33	0.044	1.000
Zn	34.41 ± 10.67	61.98 ± 15.83	0.000	2.199
Pb	4.23 ± 2.50	1.53 ± 2.57	0.006	1.059

Student *t*-tests with 95% confidence intervals (*p* = 0.05) and F-tests of equality of variances were applied to determine the significance of differences between element concentrations depending on the study area

### Markers of Hepatotoxicity and Cardiotoxicity

Biochemical indicators of hepatotoxicity and cardiotoxicity include the alanine (AlAT) and aspartate (AsAT) aminotransferase enzymes, because their molecular systems react quickly to changes in energy metabolism (Botros and Sikaris 2013). The change in their activity is of fundamental importance for early diagnosis of changes in biosynthesis and detoxification. Therefore, the next stage of the current study was to determine the activities of AlAT and AsAT in the muscle tissues. The ratio of the serum and tissue AsAT and AlAT activities is referred to as the De Ritis ratio and is used as a biomarker of hepatotoxicity. The results of our study are shown in Table 3. Our data show an increase in enzyme activities in both tissues of pigeons from the polluted region, as well as a significant increase in the De Ritis ratio, which may accompany pathological alterations in metabolism.

**Table 3:** Alanine (nmol/min mg protein) and aspartate aminotransferase (nmol/min·mg protein) activities in the skeletal muscle and cardiac tissue of pigeons sampled from the different areas (polluted area—Szpęgawa; nonpolluted area—Słupsk, Pomeranian region, Northern Poland)

Parameter	Polluted area	Nonpolluted area	*p*	*F*
*Skeletal muscles*				
AlAT	3.86 ± 0.47	4.16 ± 0.82	0.203	1.002
AsAT	5.21 ± 0.61	2.73 ± 0.61	0.000	7.02
de Ritis ratio (AsAT/AlAT)	1.349 ± 0.09	0.657 ± 0.19	0.000	20.52
*Cardiac tissue*				
AlAT	3.84 ± 0.58	3.66 ± 0.39	0.364	1.22
AsAT	4.073 ± 1.02	2.86 ± 0.49	0.000	7.25
de Ritis ratio (AsAT/AlAT)	1.061 ± 0.07	0.78 ± 0.11	0.003	5.22

Student *t*-tests with 95% confidence intervals (*p* = 0.05) and F-tests of equality of variances were applied to determine the significance of differences between enzyme activities and the de Ritis ratio depending on the types of tissues

To verify the hypothesis of the impact of the type of environments and type of tissues on the formation of the antioxidant profile in muscle tissue, we decided to evaluate these assumptions using a two-way analysis of variance. The use of Wilks’ multivariate significance test of the main effects (environment, tissue, and their combined effects) allowed us to determine statistically significant relationships for all three values. These dependencies are presented in Table 4. The MANOVA test presented in this table confirms the influence of the main factors, i.e., both the environment and tissue, and the combination of these factors on the formation of antioxidant defenses in pigeon muscle tissue. These dependencies confirm the significant role of the environments in the observed changes in the pro/antioxidant balance and markers of cardiotoxicity and hepatotoxicity in birds.

**Table 4:** Multivariate significance tests and effective hypothesis decomposition for the two types of environments (polluted, nonpolluted) and types of muscle tissues (skeletal and cardiac) for oxidative stress biomarkers and markers of cytotoxicity

Main effects	Test value	F	*p*
Environments	0.092	42.80	0.000
Tissues	0.181	19.69	0.000
Environments and tissues	0.337	8.56	0.000

For a complete analysis of the whole model of the analyzed antioxidant defense and biomarkers, we used the following test: the multiple correlation coefficients (R), the coefficient of determination (R^2^) and its corrected form reduced by random errors (R^2^ adjusted), and the F test and its significance simultaneously. The data from this group of tests are presented in Table 5. The SS test allowed us to draw the next conclusions on the role of each of the investigated data in the formation of the full statistical model in the muscle tissue. These dependencies for lipid peroxidation are as follows: ketonic derivatives of OMP > AD OMP > TAC > TBARS. The following dependencies were determined for the antioxidant enzymes: SOD > CAT > GR > GPx and for the markers of cardiotoxicity and hepatotoxicity: AsAT > AsAT/AlAT > AlAT.

**Table 5:** SS test of oxidative stress biomarkers and cytotoxicity for the whole model of results of analyses of muscle tissues and SS test for the two types of environments

Parameters	Multiple *R*	Multiple *R*^2^	Multiple adjusted *R*^2^	*F*	*p*
TBARS	0.687	0.473	0.445	17.35	0.000
Aldehydic derivatives	0.821	0.674	0.658	40.13	0.000
Ketonic derivatives	0.827	0.685	0.669	42.11	0.000
TAC	0.790	0.624	0.605	32.17	0.000
SOD	0.819	0.671	0.654	39.43	0.000
CAT	0.754	0.569	0.547	25.60	0.000
GR	0.638	0.407	0.376	13.29	0.000
GPx	0.549	0.302	0.265	8.36	0.000
AlAT	0.286	0.081	0.660	1.72	0.171
AsAT	0.822	0.677	0.034	40.57	0.000
AsAT/AlAT	0.758	0.575	0.553	26.16	0.000

In the current study, correlations between lipid peroxidation, oxidatively modified proteins, and activities of antioxidant enzymes depending on the environment and tissue studied were observed. All interdependencies presented in Table 6 are statistically significant. The results demonstrated that the effect of the environment type (polluted or nonpolluted) and the type of muscle (skeletal, cardiac) is mediated via different metabolic, elements, and redox pathways in the organism of pigeons.

**Table 6:** Correlation intergroup interdependencies between the metal levels, biomarkers of oxidative stress, and activity of aminotransferases in the feathers and muscle tissues

Relations, tissue	Polluted area	*r*	*p*	Nonpolluted area	*r*	*p*
Feathers	Al–Si	−0.577	0.015	Al-Si	−0.809	0.000
	Zn–Si	−0.720	0.001	Si-Fe	−0.715	0.003
	Ni–Cu	0.836	0.000	Cu-Fe	0.615	0.015
	Ni–Zn	0.732	0.010	Ni–Al	0.625	0.013
	Cu–Zn	0.557	0.020	Ni-Si	−0.625	0.013
				Ni-Zn	0.545	0.000
				Zn-Al	0.554	0.031
Skeletal muscle	TBARS–AlAT	0.512	0.035	TBARS–TAC	−0.599	0.018
	TBARS–de Ritis ratio	−0.585	0.014	AD OMP–CAT	0.589	0.021
	TAC–TBARS	−0.458	0.015	GR–AlAT	0.518	0.048
	TBARS–SOD	−0.587	0.023	GR–AsAT	0.607	0.016
Cardiac tissue	TBARS–TAC	0.489	0.046	TBARS–KD OMP	0.689	0.005
	AD OMP–GR	0.489	0.046	TBARS–SOD	−0.824	0.000
	SOD–AlAT	0.503	0.040	TBARS–AlAT	0.580	0.024
				TBARS–AsAT	−0.714	0.003
				TBARS– de Ritis ratio	−0.724	0.024

## Discussion

The current study demonstrated differences in the levels of heavy metals, especially Pb, in muscle tissues (skeletal and cardiac muscles) in pigeons from environments with different levels of pollution. The research objective of this study was to determine the relationship between biomarkers of lipid peroxidation, oxidatively modified proteins, activities of antioxidant enzymes, and heavy metals in soil and feather of birds. The present study demonstrated the impact of the polluted environment and type of muscle tissues (skeletal and cardiac) on the formation of an effective adaptive profile of redox mechanisms depending on the hepatotoxicity and cardiotoxicity markers analyzed in this study.

It should be noted that most research on pigeons in contaminated environments has been performed on tissue types that show biotransformation of heavy metals in such conditions. In most cases, these are blood (Kurhalyuk et al. 2007; Kouddane et al. 2016), lung (Pei et al. 2017; Cui et al. 2018), liver and kidney tissue (Schilderman et al. 1997; Liu et al. 2010; Cui et al. 2013, 2016), and feathers (Dauwe et al. 2003; Frantz et al. 2012; Lodenius and Solonen 2013; Grúz et al. 2019). However, there are still few studies conducted on muscle tissue, which is characterized by high functional plasticity of the adaptation mechanisms, behavioral reactions, and adaptability. To a large extent, this is the case in skeletal muscle and cardiac tissues, where energy exchange has functional features of redox reactions and is related to the physical condition of birds. For this reason, our research targeted this issue, which is poorly represented in the literature.

There are several novel findings in our study. First, in our environmental investigation model, the preferential Pb exposure in our results indicated a twofold higher level of oxidatively modified proteins, namely aldehydic and ketonic derivatives, compared with TBARS products, which may be a specific mechanism of the effects of the polluted environment on the physiological state of skeletal muscles in birds. The increase in the content of aldehydic and ketonic derivatives of oxidatively modified proteins only in the skeletal muscular tissue (no such effects were established in the cardiac tissue) together with the high level of cell toxicity biomarkers indicated effects induced by lead toxicity. Several investigators reported that lead-induced necrotic changes with simultaneous mitogenic activity; however, it did not induce significant DNA damage in the liver (Narayana and Al-Bader 2011; Narayana and Raghupathy 2012). Hepatocytes display necrosis-related changes in the lead-nitrate-exposed liver (Narayana and Raghupathy 2012). Qualitatively, necrotic changes, such as small- to large-sized cytoplasmic vacuoles often displacing other organelles, a decrease in hepatocyte microvilli, degeneration of mitochondria, and vacuolar encroachment of nuclei and dilatation of sinusoids were observed as well (Narayana and Al-Bader 2011). The ability of lead to enhance lipid peroxidation may be attributed to its indirect effect on free radical scavenging enzymes in tissues and not to its direct effect on lipid peroxidation, because lead does not participate in the oxidation–reduction cycle (Flora et al. 2008).

Fe, Cu, and Zn levels were higher in feather samples of pigeons from the nonpolluted environment than in the polluted area (Table 2). Probably, the differences between areas may be due to differences in geochemistry between the sites, because data on Zn in the soils support it. On the other hand, Fe levels in soil samples were higher in a polluted environment, while Cu levels were statistically nonsignificant between areas (Table 1). Iron is an essential element for a number of crucial processes, i.e., hemoglobin and myoglobin transport, storage of oxygen in mammals, electron transfer support in a variety of iron-sulfur protein or cytochrome reactions, activation, and catalysis of a wide range of reactions (Sánchez et al. 2017). On the other hand, excessive Fe accumulation (hemochromatosis) in tissues has been manifested by cellular necrosis and fibrosis, often with associated functional changes in the liver, the heart, and endocrine tissues related to increased prevalence of infections, neoplasia, hepatopathy, cardiomyopathy, arthropathy, endocrinopathies, and neurodegenerative disorders (Whiteside et al. 2004). Cu is an essential element, and it is important for the function and structure of proteins and in electron transfer reactions (Balsano et al. 2018). Cu as a catalytic cofactor is required for the activities of CuZn-SOD and ceruloplasmin. Cu deficiency can decrease the activities of catalase and selenium-dependent glutathione peroxidase (Se-GPx) and alter metallothionein, nonprotein thiol, glutathione (Uriu-Adams and Keen 2005). Zn is another essential element that is a cofactor of more than 200 enzymes functioning in diverse physiological processes, including immunity, antioxidant abilities, reproductive activity, growth and development, and epigenetic processes (Huang et al. 2019; Yasmeen et al. 2019).

Second, among the four antioxidant enzymes (SOD > CAT > GR > GPx) analyzed depending on the type of environments and tissues, superoxide dismutase was shown to play the main role in the antioxidant defense. Antioxidant enzymes can also protect cellular compounds against damage induced by free radicals at a heavy metal impact. Superoxide dismutases (SOD), catalase (CAT), and glutathione peroxidases (GPx) are important antioxidant enzymes (Koivula et al. 2011; Pizzino et al. 2017). SOD decomposes superoxide radicals (O_2_^•−^) and produces H_2_O_2_. H_2_O_2_ is subsequently removed to water by CAT in the peroxisomes or by GPx oxidizing GSH in the cytosol (Koivula and Eeva 2010). The activities of these enzymes have been used to assess oxidative stress in cells.

Lead antagonizes biological systems by attracting strong oxidizers (O_2_) and active metals (sodium, potassium) and inevitably disrupting normal cellular metabolism. This interference leads to the generation of highly reactive oxygen species (ROS), namely superoxides (O_2_^·–^), hydrogen peroxide (H_2_O_2_), hydroxyl radicals (OH^–^), and lipid peroxides causing damage to cellular components including proteins, membrane lipids, and nucleic acids. One of the proposed mechanisms of lead toxicity is the contribution of lead-induced oxidative stress to the deleterious effects by disruption of the delicate prooxidant/antioxidant balance in mammalian cells. Lead has been found to produce a wide range of toxic biochemical effects, besides behavioral dysfunction in man and experimental animals (Williams et al. 2018).

Many studies have shown that lead can alter antioxidant activities by inhibiting functional SH groups in several enzymes, such as SOD, CAT, and GPx, due to its high affinity for sulfhydryl (SH) groups in these enzymes (Koivula et al. 2011). GPx, CAT, and SOD depend on various essential trace elements for proper molecular structure and activity; therefore, these antioxidant enzymes are potential targets for lead toxicity (Rehman et al. 2011; Williams et al. 2018). Moreover, when the balance between ROS production and antioxidant defenses is broken, enzymes may be exhausted and their concentration is depleted. In our study, the enzyme activities associated with the first and second lines of antioxidant defenses, i.e., SOD and CAT activities, were drastically reduced. The lowest SOD activity was observed for the skeletal muscles of pigeons from the polluted area. CAT activity exhibited a similar tendency to that found for SOD, i.e., a tendency to decrease in the pigeons from the polluted environment. It should be noted that the decrease in the catalase activity in the skeletal muscle and cardiac tissue (sixfold and threefold, respectively) of pigeons from the polluted area may indicate induction of utilization of hydrogen peroxide in the conditions of lead poisoning.

Lead-induced oxidative stress contributes to the pathogenesis of lead poisoning by disruption of the delicate prooxidant/antioxidant balance in mammalian cells. Production of ROS is increased after lead treatment in vitro studies. In vivo studies suggest that lead exposure causes the generation of ROS and alteration of antioxidant defense systems in animals and occupationally exposed workers. The mechanisms of lead-induced oxidative stress include the effect of lead on the membrane, DNA, and antioxidant defense systems of cells. At low to high doses of lead exposure, there are different responses of lead-induced oxidative stress in various target sites, including lung, blood vessels, testes, sperm, liver, and brain in epidemiological and animal studies (Hsu and Guo 2002).

Third, metals can cause oxidative stress by increasing the formation of ROS when the amounts of antioxidants are insufficient to defend against the growing amounts of free radicals (Pizzino et al. 2017; Koim-Puchowska et al. 2020). The effects of other metals on the induction of oxidative stress in the skeletal muscles shown in the correlation analysis also should be noted. Correlative dependences were observed in the skeletal muscle tissue of pigeons from the nonpolluted environment, i.e., Al and aldehydic derivatives (*r* =  − 0.685, *p* = 0.005), Al and ketonic derivatives (*r* = 0.664, *p* = 0.007), Ni and aldehydic derivatives (*r* = 0.564, *p* = 0.029), Ni and ketonic derivatives (*r* = 0.574, *p* = 0.025), Fe and GPx (*r* =  − 0.535, *p* = 0.040), and Fe and TAC (*r* =  − 0.527, *p* = 0.044). Correlative dependences were also observed in the skeletal muscle tissue of pigeons from the polluted environment, i.e. Cu and aldehydic derivatives (*r* =  − 0.581, *p* = 0.014), Cu and ketonic derivatives (*r* =  − 0.567, *p* = 0.018), and Cu and GR activity (*r* = 0.578, *p* = 0.015; Table 6).

In the cardiac tissue of pigeons from the nonpolluted environment, the following correlative dependences were observed: Fe and SOD activity (*r* = 0.530, *p* = 0.042) and Pb and AlAT activity (*r* = 0.518, *p* = 0.048). Correlative dependences were observed in the cardiac tissue of pigeons from the polluted environment, i.e., TBARS and Si (*r* = 0.486, *p* = 0.048) and Pb and GPx activity (*r* =  − 0.561, *p* = 0.019; Table 6). The level of these trace elements has a significant impact on the activity of antioxidant enzymes and biomarkers of cytotoxicity, and thus on the defense against oxidative stress. Even a small change in the level of trace elements in the tissue causes disturbances in their metabolism, leading to the occurrence of many disorders. Similar effects were demonstrated by our scientific team (Kamiński et al. 2009) and other authors in different models of birds living in environments with different levels and types of contamination (Wołonciej et al. 2016).

Fourth, our results confirm the concept of the recalculation of the De Ritis coefficient (AsAT/AlAT) in both types of muscle tissues. These enzymes represent the equilibrium between the normal turnover of hepatocytes and the clearance of the enzymes from plasma (Botros and Sikaris 2013). The tests used to describe the profile of selected biomarkers of cytotoxicity demonstrated a key role of AsAT in the formation of the full statistical model. Our data showed an increase in enzyme activity in both tissues of pigeons from the polluted region and a statistically significant increase in the De Ritis ratio, which may show pathological changes in the metabolic processes. Thus, not only the dynamics of the activity of these two enzymes but primarily the ratio of their activity is important. Our data confirm this concept. AlAT and AsAT activity is commonly requested blood tests in liver and heart diseases. The release of AlAT and AsAT from hepatocytes to the bloodstream represents hepatocellular damage caused by heavy metals.

## Conclusions

Metal-related oxidative stress in muscle tissue (skeletal muscle and cardiac tissues) is a complex of different biomolecular mechanisms involved in adaptive reactions in pigeons living in environments with different levels of metal bioaccumulation, as shown in the Pomerania region (Northern Poland). The current study demonstrated the impact of the environment with preferential high Pb contamination in soil and pigeon feathers collected in Szpęgawa village located near the A1 motorway on the formation of adaptive redox mechanisms in the skeletal muscle and cardiac tissues of pigeons. The increase in the intensity of lipid peroxidation (estimated by the TBARS level) was accompanied by an increase in oxidative modifications of proteins (aldehydic and ketonic derivatives) with a significant decrease in antioxidant enzyme activities (SOD, CAT, and GR) in the muscle tissue of pigeons. The changes in enzyme activities were dependent on the type of muscle tissues (skeletal muscle and cardiac tissues). Our results confirm the concept of the recalculation of the De Ritis coefficient (AsAT/AlAT) in both types of muscles, which indicates the tendency to cardio- and hepatocellular damage and toxicity caused by heavy metals from polluted environments.
